# Natural Variation in the *Drosophila melanogaster* Clock Gene *Period* Modulates Splicing of Its 3′-Terminal Intron and Mid-Day Siesta

**DOI:** 10.1371/journal.pone.0049536

**Published:** 2012-11-13

**Authors:** Kwang Huei Low, Wen-Feng Chen, Evrim Yildirim, Isaac Edery

**Affiliations:** 1 Center for Advanced Biotechnology and Medicine, Rutgers University, Piscataway, New Jersey, United States of America; 2 Department of Molecular Biology and Biochemistry, Center for Advanced Biotechnology and Medicine, Rutgers University, Piscataway, New Jersey, United States of America; Yale School of Medicine, United States of America

## Abstract

*Drosophila melanogaster* exhibits circadian (≅24 hr) regulated morning and evening bouts of activity that are separated by a mid-day siesta. Increases in daily ambient temperature are accompanied by a progressively longer mid-day siesta and delayed evening activity. Presumably, this behavioral plasticity reflects an adaptive response that endows *D. melanogaster* with the ability to temporally optimize daily activity levels over a wide range of physiologically relevant temperatures. For example, the shift in activity towards the cooler nighttime hours on hot days might minimize the risks associated with exposure to mid-day heat, whereas on cold days activity is favored during the warmer daytime hours. These temperature-induced shifts in the distribution of daily activity are partly based on the thermal sensitive splicing of an intron found in the 3′ untranslated region (UTR) of the circadian clock gene termed *period* (*per*). As temperature decreases, splicing of this 3′-terminal intron (termed dmpi8) is gradually increased, which is causally linked to a shorter mid-day siesta. Herein we identify several natural polymorphisms in the *per* 3′ UTR from wild-caught populations of flies originating along the east coast of the United States. Two non-intronic closely spaced single nucleotide polymorphisms (SNPs) modulate dmpi8 splicing efficiency, with the least efficiently spliced version associated with a longer mid-day siesta, especially at lower temperatures. Although these SNPs modulate the splicing efficiency of dmpi8 they have little to no effect on its thermal responsiveness, consistent with the notion that the suboptimal 5′ and 3′ splice sites of the dmpi8 intron are the primary *cis*-acting elements mediating temperature regulation. Our results demonstrate that natural variations in the *per* gene can modulate the splicing efficiency of the dmpi8 intron and the daily distribution of activity, providing natural examples for the involvement of dmpi8 splicing in the thermal adaptation of behavioral programs in *D. melanogaster*.

## Introduction

Daily wake-sleep cycles in animals are governed by networks of cell-based circadian (≅24 hr) ‘clocks’ or pacemakers located in the brain. Similar to many diurnal animals, the daily distribution of activity in *Drosophila melanogaster* can exhibit a bimodal pattern with clock-controlled morning and evening peaks separated by a mid-day ‘siesta’ [1]. Increases in average daily temperature are accompanied by a gradual delay in the onset of the evening bout of activity and a more robust mid-day siesta [2], [3], [4], [5]. It is thought that this thermally regulated behavioral plasticity endows *D. melanogaster* with the ability to adapt to seasonal changes in temperature [3], [5]. For example, the increased mid-day activity of flies on cold days might be an adaptive response to maximize activity during the warmer daytimes hours, whereas suppressing mid-day activity with a concomitant shift towards the cooler nighttime hours on warm days might minimize the risks associated with unnecessary energy expenditure during the hot mid-day sun. We showed that this temperature-dependent behavioral adaptation is at least partially controlled by thermosensitive splicing of a 3′-terminal intron from the *Drosophila melanogaster period* (*dper*) transcript [3], [5], which is a key circadian clock factor known for encoding species-specific circadian behavioral programs in this species [3], [6], [7], [8]. The removal of a short intron in the 3′ untranslated region (UTR) of d*per* RNA, named dmpi8 (*D. melanogaster per* intron 8), is inefficient at warmer temperatures, which attenuates the daily accumulation of *dper* mRNA, somehow leading to delayed evening activity and longer mid-day siesta [3], [5].

Temperature dependent splicing of dmpi8 was shown to result from suboptimal 5′ and 3′ splicing signals (ss), suggestion that splice site recognition/binding by the spliceosome to dmpi8 is inefficient at higher temperatures [3]. Most notably, transgenic flies whereby the dmpi8 5′ and 3′ss were optimized exhibited near total removal of the dmpi8 intron at all temperatures and displayed less robust mid-day siestas compared to their wildtype control transgenics [3]. In this study, we sought to investigate the possibility of genetic variability harbored by natural populations of *D. melanogaster* that might affect the splicing efficiency of the dmpi8 intron and hence daily activity patterns. As an initial test case, we examined numerous independent isofemale lines of *D. melanogaster* that were originally established by capturing flies along the Atlantic coast of the United States [9]. In all the flies we examined from this collection the *dper* gene contains a dmpi8 intron with the identical suboptimal 5′ and 3′ splice sites as originally reported [3]. However, we identified several natural polymorphisms in the *dper* 3′ UTR. Two closely spaced single nucleotide polymorphisms (SNPs) that are far removed from intronic sequences modulate dmpi8 splicing efficiency and daily activity patterns, whereby more efficient splicing is causally linked to a less robust mid-day siesta and earlier evening activity, especially at cooler temperatures. Although these natural variants modulate the average daily splicing efficiency of the dmpi8 intron, they have little to no effect on the thermal responsiveness of this splicing event. Thus, while the suboptimal 5′ and 3′ss of dmpi8 are critical for thermosensitive splicing, non-intronic sequences can tweak the splicing efficiency, revealing a link between natural polymorphisms in clock genes and heritable differences in the thermal adaptation of behavioral programs in animals.

## Results

### Identification of Natural Polymorphisms in the 3′ UTR of *dper*

A role for dmpi8 splicing in regulating daily activity in *D. melanogaster* was partly supported by generating transgenic flies whereby we altered the splicing efficiency of the dmpi8 intron by engineering changes to the sequences of key splicing recognition signals [3], [5]. An important finding is that the temperature sensitive splicing of dmpi8 is based upon suboptimal 5′ and 3′ss [3]. In this study we sought to investigate the possibility of natural variants in the *dper* 3′ UTR that might affect dmpi8 splicing and daily activity. As an initial test case we used a previously characterized collection of *D. melanogaster* that contains several independent isofemale lines from each of 10 populations along a latitudinal cline in the eastern United States that stretches from Florida to Vermont [9]. We sequenced the *dper* 3′ UTR from at least 5 isofemales lines from each of the 10 different locations.

**Figure 1:**
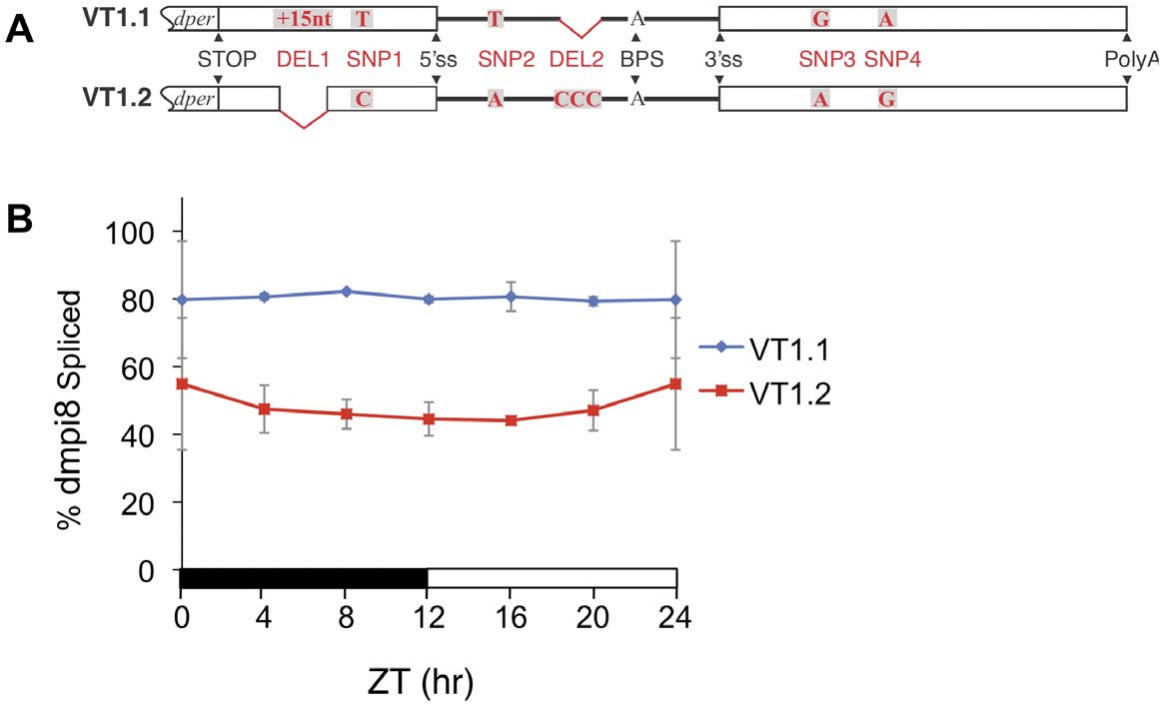
Splicing of the dmpi8 intron is more efficient in the VT1.1 compared to VT1.2 inbred flies. **A.** Schematic representation of the *dper* 3′ UTR from VT1.1 and VT1.2 flies, showing the six polymorphisms identified in this study (highlighted in red); STOP, *dper* translation stop signal; DEL1, in the VT1.2 haplotype is missing 15 nt compared to VT1.1; 5′ss, 5′ splice site; BPS, branchpoint; 3′ss, 3′ splice site; polyA, 3′ cleavage/polyadenylation site. **B.** Flies were collected at the indicated times during 12∶12LD at 18°C, head extracts prepared and the proportion of *dper* transcripts with dmpi8 spliced is shown for VT1.1 (blue) and VT1.2 (red) flies. The values shown are an average of three independent experiments. Similar results whereby the daily splicing of dmpi8 is more efficient in VT1.1 compared to VT1.2 flies were also obtained at 29°C (data not shown). For each time-point, the dmpi8 splicing efficiency in VT1.1 flies is significantly different from that in VT1.2 flies (ANOVA, p < 0.01).

All the *dper* 3′ UTRs we analyzed from these natural populations have a similarly sized dmpi8 intron (86 or 89 bp in length) approximately 100 bp downstream of the translation stop codon, with identical suboptimal 5′ and 3′ss, including the same branchpoint signal (BPS) (Fig. 1A and data not shown). This is consistent with the observations that thermosensitive splicing of dmpi8 and temperature dependent changes in daily activity patterns are a ubiquitous feature of *D. melanogaster*, exhibited by a wide range of laboratory strains and natural populations from diverse geographical locations ([3], and data not shown). Although the key splicing signals of the dmpi8 intron appear highly conserved in *D. melanogaster*, we identified two major haplotypes of the *dper* 3′ UTR that differ from each other by six polymorphisms (Fig. 1A and data not shown). Four of the sequence variations are single nucleotide polymorphisms (SNPs; herein termed SNP1, SNP2, SNP3 and SNP4) and the other two are deletions/insertions (termed DEL1 and DEL2). SNP2 and DEL2 are located within the dmpi8 intron, whereas the rest are situated at least 45 bp away from the intron and are thus unlikely to directly affect the strengths of the key splicing recognition signals, most notably the 5′ss, 3′ss and BPS (Fig. 1A).

**Figure 2:**
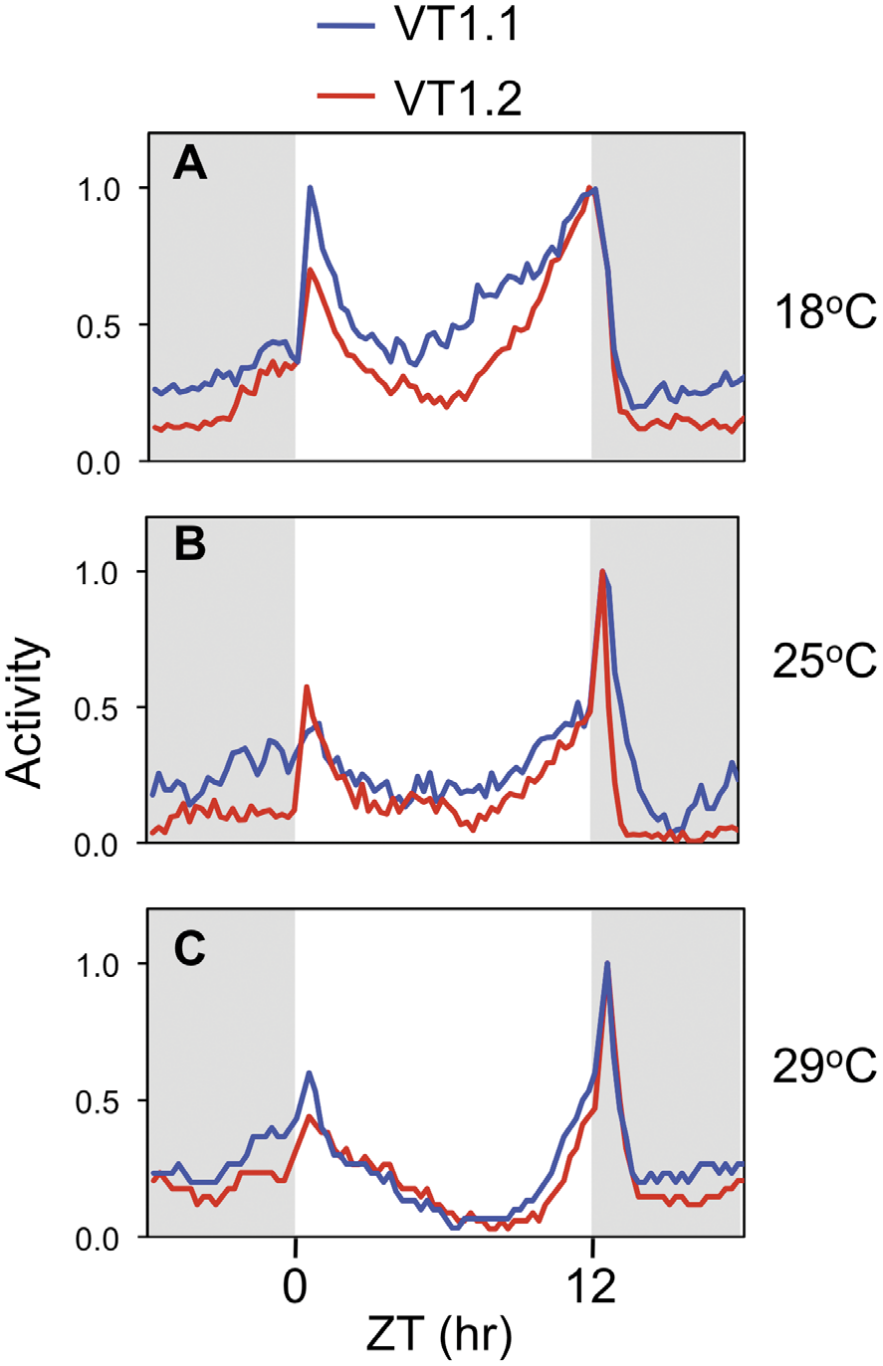
Mid-day siesta is less robust and evening activity onset is earlier in the VT1.1 compared to VT1.2 inbred flies. Flies were exposed to 12∶12LD cycles for 5 days at 18° (A), 25° (B), or 29°C (C), and then kept in constant darkness (DD) for 7 days. Shown are the locomotor activity profiles for VT1.1 (blue) and VT1.2 (red) flies during 12∶12LD. Data recorded during the last three days of 12∶12LD for individual flies were combined to obtain the group averages shown. To facilitate comparison, the peak value for evening activity for each genotype was set to 1 and all other values normalized. Grey shading, lights-off.

Comparisons of DNA sequences did not reveal any obvious latitudinal or inter- population differences in the relative distributions of the two *dper* 3′ UTR haplotypes (data not shown). With regards to daily activity patterns, there was much intra-population variability between independent isofemale lines from the same location making it difficult to determine if there are significant differences in the daily distributions of activity between the different geographical populations and/or haplotypes (data not shown). Because of the high intra-population variability in the daily distribution of activity we sought to establish sub-lines from a single isofemale line that would be homozygous for each haplotype in the hopes of providing a more isogenic platform to detect possible relationships between dmpi8 splicing efficiency and daily behavior profiles. In this regard, we used the VT97.1 isofemale line from Vermont [9], in which both haplotypes were observed with approximately equal distribution in the population (data not shown). Using single-mate crossing and DNA sequencing to verify genotypes, we established two sub-lines termed VT1.1 and VT1.2 that are homozygous for either of the haplotypes (Fig. 1A and data not shown). Unfortunately, only a limited number of behavioral and molecular results were obtained with these inbred lines (e.g., Figs. 1 and 2; discussed below) because they and our entire collection of isofemale lines from this United States collection perished during an incubator malfunction (to the best of our knowledge, these lines are no longer extant in other laboratories). Importantly however, similar results were obtained in transgenic fly models representative of the same *dper* 3′ UTR haplotypes (see below).

### The VT1.1 and VT1.2 Strains Exhibit Differences in dmpi8 Splicing Efficiency and Daily Activity Patterns

We first sought to determine whether the splicing efficiency of the dmpi8 intron in the VT1.1 and VT1.2 inbred flies differ, which could suggest a possible role for one or more of the *dper* 3′ UTR polymorphisms in regulating intron removal. Prior work showed that at higher temperatures the splicing efficiency of dmpi8 exhibits a moderate daily oscillation due to increased daytime repression by light and the clock, whereas dmpi8 splicing efficiency is relatively constant throughout a daily cycle at lower temperatures, such as 18°C [2], [3], [4], [5]. Flies were kept at 18°C and entrained (synchronized) under standard conditions of 12 hr light followed by 12 hr dark cycles [12∶12LD, where zeitgeber time 0 (ZT0) is lights-on and ZT12 is lights-off]. Subsequently, flies were collected at different times throughout a daily cycle, head extracts prepared and the dmpi8 splicing efficiency measured using RT-PCR as previously described [3], [4]. The results clearly indicate that throughout a daily cycle, the splicing efficiency of dmpi8 is significantly higher in VT1.1 flies compared to VT1.2 flies (ANOVA, p<0.01 for each time point; Fig. 1B).

Having shown that the dmpi8 splicing efficiency differs in VT1.1 and VT1.2 flies, we next measured locomotor activity rhythms. *D. melanogaster* exhibit daily rhythms in locomotor activity that are regulated by the circadian timing system [1]. Under standard laboratory conditions of daily 12 hr light/12 hr dark cycles, two clock-controlled peaks of activity have been documented in *D. melanogaster*; i.e., a ‘morning’ peak (centered around the dark-to-light transition at ZT0) and an ‘evening’ peak (centered around the light-to-dark transition at ZT12) that are separated by a mid-day ‘siesta’ or dip in activity levels [1], [5], [10]. Overlaying the clock regulation of daily activity are more acute responses caused by the light/dark transitions that evoke transient increases in activity (sometimes referred to as ‘startle’ responses or masking effects) [11]. As temperature increases the onset of the evening bout of activity is progressively delayed and the mid-day siesta increases in length and is more robust [5].

VT1.1 and VT1.2 flies were kept at different temperatures and entrained under standard 12∶12LD cycles for several days to obtain daily activity profiles, followed by at least one week of complete darkness (DD) to determine the endogenous period of the daily activity rhythms. Both the VT1.1 and VT1.2 inbred lines exhibit the normal trend towards longer mid-day siesta and later evening activity as daily ambient temperature increases (Fig. 2 and Table 1). However, in VT1.1 flies the onset of evening activity is advanced and the mid-day siesta is shorter compared to VT1.2 flies (Fig. 2 and Table 1). Differences in mid-day siesta and the timing of evening activity between VT1.1 and VT1.2 flies are most readily observed at 18°C (Fig. 2). The more dramatic behavioral differences at colder temperatures between flies exhibiting differential dmpi8 splicing efficiencies are consistent with prior work [3], [5]. At higher temperatures light has more potent effects on directly suppressing activity, likely minimizing or negating contributions from changes in dmpi8 splicing efficiency (e.g., [5], [12]). Thus, although the correlation between dmpi8 splicing efficiency and length of mid-day siesta are mainly observed at lower temperatures, the results using these inbred lines are consistent with our earlier findings using transgenic flies whereby those bearing a version of dmpi8 that is more efficiently spliced exhibit shorter mid-day siestas and earlier onsets of evening activity [3]. No significant period difference in free-running behavioral rhythms were observed between the VT1.1 and VT1.2 at all temperatures tested (Table 2), in agreement with prior results indicating that daily changes in the splicing efficiency of dmpi8 modulate the distribution of daily activity but do not alter clock speed [3], [5]. In addition to a shorter mid-day siesta, VT1.1 flies also exhibit relatively higher nighttime activity levels (Fig. 2), which could arise from other strain-specific genetic differences (see below). In summary, the results indicate that natural populations of *D. melanogaster* have polymorphisms in the *dper* 3’ UTR, and suggest that some or all of these molecular differences regulate the overall daily efficiency of dmpi8 splicing, leading to heritable phenotypic differences in the distribution of daily activity.

**Table 1:** Timing of daily activity and mid-day siesta in inbred and transgenic flies.

Genotype^a^	Temperature		Morning offsetc,d	Evening onset^c^.^e^	Siesta^f^
	(°C)	n^b^	(hr ± sem)	(hr ± sem)	(hr ± sem)
VT1.1 (inbred)	18	81	2.9 ± 0.1	7.9 ± 0.2	5.0 ± 0.2*
	29	52	2.7 ± 0.2	9.6 ± 0.2	6.9 ± 0.3*
VT1.2 (inbred)	18	90	2.3 ± 0.2	8.5 ± 0.1	6.2 ± 0.2
	29	54	2.3 ± 0.2	10.2 ± 0.2	8.0 ± 0.3
*w per* ^01^;p{VT1.1}	18	81	2.3 ± 0.2	6.4 ± 0.2	4.1 ± 0.2*
	25	139	2.1 ± 0.2	7.6 ± 0.2	5.5 ± 0.2
	29	207	1.8 ± 0.1	10.1 ± 0.1	8.4 ± 0.2
*w per* ^01^;p{VT1.2}	18	138	2.3 ± 0.2	7.2 ± 0.1	4.9 ± 0.2
	25	119	2.6 ± 0.2	8.3 ± 0.2	5.8 ± 0.2
	29	182	1.2 ± 0.2	10.0 ± 0.1	8.9 ± 0.2
*w per* ^01^;p{VT1.1-SNP3}	18	82	2.9 ± 0.2	6.2 ± 0.2	3.3 ± 0.2
	25	161	2.5 ± 0.1	8.4 ± 0.2	5.9 ± 0.2
	29	32	1.9 ± 0.3	10.1 ± 0.3	8.2 ± 0.3
*w per* ^01^;p{VT1.1-SNP4}	18	154	2.5 ± 0.1	6.4 ± 0.2	3.9 ± 0.2
	25	161	2.4 ± 0.1	8.2 ± 0.1	5.8 ± 0.2
	29	16	1.7 ± 0.1	10.2 ± 0.1	8.5 ± 0.2
*w per* ^01^;p{VT1.1-SNP3/4}	18	38	1.8 ± 0.3	6.8 ± 0.2	5.0 ± 0.3#
	25	116	2.4 ± 0.2	9.1 ± 0.1	6.7 ± 0.3#
	29	16	1.1 ± 0.2	11.1 ± 0.1	10.0 ± 0.2#

a Young male flies were exposed to five days of 12∶12LD at the indicated temperature. For each genotype, the last three days worth of activity was averaged for each individual fly and then a group average was determined. For the transgenic flies, data from at least three independent lines was used.

b n, number of flies used to calculate the values shown and that survived the entire testing period.

c Values denote zeitgeber time, whereby ZT0 is defined as lights-on.

d Morning offset is defined as the ZT time during the morning activity downswing when 50% of peak morning activity was attained.

e Evening onset is defined as the ZT time during the evening activity upswing when 50% of peak evening activity was attained.

f Siesta time is defined as the length of time between 50% of morning offset and 50% of evening onset. *, significantly different (p < 0.01, Student’s *t-test*) value for VT1.1 inbred or transgenic compared to VT1.2 counterpart. #, significantly different (p < 0.01, Student’s *t-test*) value for p{VT1.1-SNP3/4} compared to either p{VT1.1-SNP3} or p{VT1.1-SNP4}.

**Table 2:** Circadian values for daily activity rhythms in inbred and transgenic flies.

Genotype^a^	Temperature		Period	Rhythmicty^c^	Power^d^
	(°C)	n^b^	(hr ± sem)	(%)	(± sem)
VT1.1 (inbred)	18	126	24.2 ± 0.1	84.9	72.0 ± 3.1
	29	126	24.4 ± 0.1	96.1	68.4 ± 3.8
VT1.2 (inbred)	18	127	24.2 ± 0.1	85.7	82.1 ± 3.4
	29	116	24.5 ± 0.1	88.8	85.4 ± 3.6
*w per* ^01^;p{VT1.1}	18	127	23.9 ± 0.1	92.1	81.3 ± 6.0
	25	126	23.9 ± 0.1	93.0	83.1 ± 5.9
	29	29	23.8 ± 0.1	96.4	96.6 ± 6.0
*w per* ^01^;p{VT1.2}	18	126	23.9 ± 0.1	94.4	92.0 ± 5.2
	25	126	24.1 ± 0.1	92.9	81.6 ± 4.6
	29	15	23.8 ± 0.1	100.0	100.3 ± 4.3
*w per* ^01^;p{VT1.1-SNP3}	18	63	23.5 ± 0.1	84.1	66.2 ± 4.0
	25	64	23.9 ± 0.1	92.2	80.2 ± 4.8
	29	32	23.6 ± 0.1	96.9	87.5 ± 5.6
*w per* ^01^;p{VT1.1-SNP4}	18	62	23.5 ± 0.1	85.5	88.4 ± 4.4
	25	63	23.7 ± 0.1	95.2	96.6 ± 4.2
	29	32	23.6 ± 0.1	100.0	101.6 ± 4.0
*w per* ^01^;p{VT1.1-SNP3/4}	18	63	23.5 ± 0.1	92.1	89.1 ± 4.1
	25	59	23.8 ± 0.1	96.6	93.5 ± 4.7
	29	29	23.5 ± 0.1	100.0	102.3 ± 5.8

a Young male flies were exposed to five days of 12∶12LD at the indicated temperature, followed by 7 days in continuous darkness (DD). For each genotype, activity data from DD was averaged for each individual fly and then a group average was determined. For the transgenic flies, data from at least three independent lines was used.

b n, number of flies used to calculate the values shown and that survived the entire testing period.

c Flies with a power value of greater than 10 and period ≥ 20 and ≤ 30, were defined as rhythmic.

d Power is a measure of the strength or amplitude of the rhythm in arbitrary units.

### Transgenic Flies Carrying the VT1.1 and VT1.2 Haplotypes Recapitulate the Differences in dmpi8 Splicing and the Daily Activity Profiles Observed in Natural Populations

To eliminate the possibility that genetic background difference (i.e., other genetic loci besides the *dper* 3′ UTR) between the VT1.1 and VT1.2 inbred lines underlie the differences in dmpi8 splicing efficiency and/or the daily distribution of locomotor activity, we generated transgenic flies that carry *dper* versions with either the VT1.1 or VT1.2 3′ UTR. The different variants were engineered into a previously characterized vector that contains a 13.2-kb *dper* genomic fragment and which rescues wildtype behavioral rhythms in the arrhythmic *per*-null *per* ^01^ genetic background [13], [14]. Thus, these flies contain two different versions of the *dper* gene that can generate transcripts–1) the transgene derived version which is the only functional copy of *dper*, and 2) the endogenous non-functional *per* ^01^ version. Several independent lines of transgenic flies bearing the different transgenes (herein referred to as p{VT1.1} and p{VT1.2}) were obtained and evaluated for circadian functionality in the *w per* ^01^ genetic background (*w per* ^01^;p{VT1.1} and *w per* ^01^;p{VT1.2}; herein, more simply termed p{VT1.1} and p{VT1.2}, respectively). We used transcript specific primers to measure dmpi8 splicing efficiency from either the transgene or the endogenous *per* ^01^ gene.

**Figure 3:**
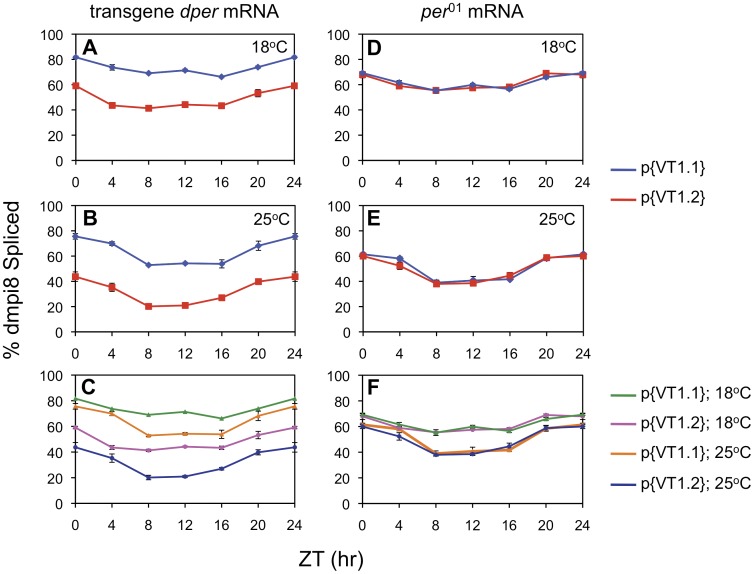
Splicing of the dmpi8 intron in transgenic flies bearing the VT1.1 and VT1.2 *dper* 3 ′ **UTR haplotypes is similar to that observed in the inbred lines.** (A, B, D and E) Shown is the proportion of *dper* mRNA with the dmpi8 intron spliced for the p{VT1.1} (blue) and p{VT1.2} (red) transgenic flies, for either transgene-derived (A, B) or *per* ^01^-derived (D, E) *dper* transcripts. (C, F) Shown is the proportion of *dper* mRNA with the dmpi8 intron spliced for the p{VT1.1} and p{VT1.2} transgenic flies at either 18° or 25°C (as indicated, right of panels), for either transgene-derived (C) or *per* ^01^-derived (F) *dper* transcripts. Note that the overall daily splicing efficiency of dmpi8 in both p{VT1.1} and p{VT1.2} flies is reduced at warmer temperatures. For each panel, the results shown are an average of at least three independent experiments. At all temperatures tested, the splicing efficiency for the dmpi8 intron in p{VT1.1} flies is significantly different from that in p{VT1.2} flies (ANOVA, p < 0.01). ANOVA analysis also showed significant temperature effects (p < 0.01) on dmpi8 splicing efficiency for both p{VT1.1} and p{VT1.2} flies.

At all temperatures analyzed, the daily splicing efficiency of dmpi8 from transgene-derived transcripts is significantly higher in p{VT1.1} flies compared to p{VT1.2}) (Fig. 3, A and B; and data not shown). The daily mean splicing efficiency of dmpi8 was increased at lower temperatures for both the VT1.1 and VT1.2 versions (Fig. 3C), indicating that while the different polymorphisms in the *dper* 3′UTR modulate splicing efficiency, they do not abrogate the thermal sensitivity of dmpi8 splicing. This is consistent with our prior work showing that the thermal sensitivity in dmpi8 splicing is based on suboptimal 5′ and 3′ss, which are identical in the VT1.1 and VT1.2 haplotypes (Fig. 1A) [3]. Also consistent with prior work (and as noted above), at higher temperatures the daily splicing efficiency of dmpi8 exhibits a more prominent daily oscillation as a result of stronger mid-day to early-night repression by the clock and light [2], [4], [5]. Thus, when comparing the daily dmpi8 splicing curves at low and high temperatures for each genotype, the largest differences occur between mid-day and early night (Fig. 3C). In this regard, it is possible that splicing of the dmpi8 intron in p{VT1.2} flies is relatively more sensitive to inhibition by a combination of temperature, light and clock compared to p{VT1.1} flies (Fig. 3C).

To further support the notion that *cis*-acting elements in the *dper* 3′ UTR are responsible for the differences in the dmpi8 splicing efficiency observed in the p{VT1.1} and p{VT1.2} transgenic flies, we also measured dmpi8 splicing efficiency from the endogenous *per* ^01^ transcripts. Indeed, for each temperature analyzed, the dmpi8 splicing efficiency from the endogenous *per* ^01^ transcripts was virtually identical in p{VT1.1} and p{VT1.2} flies (Fig. 3, D and E). As previously shown, *per* ^01^ mRNAs also exhibit thermally regulated dmpi8 splicing efficiency (Fig. 3F) [2], [3], [4], [5].

**Figure 4:**
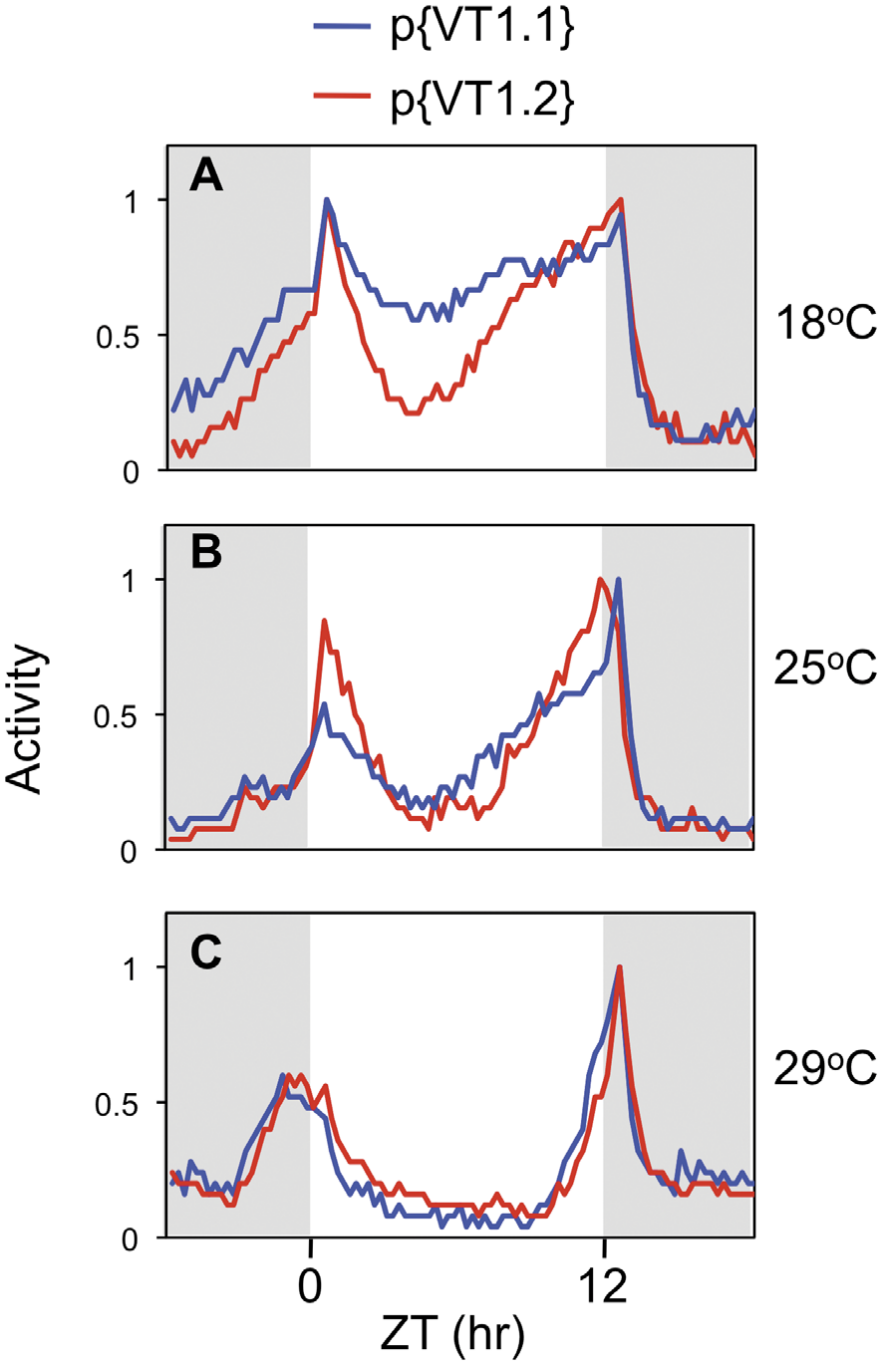
More prominent mid-day siesta in p{VT1.2} compared to p{VT1.1} transgenic flies, similar to their inbred counterparts. Flies were exposed to 12∶12LD cycles for 5 days at 18° (A), 25° (B), or 29°C (C), and then kept in constant darkness (DD) for 7 days. Shown are the locomotor activity profiles for p{VT1.1} (blue) and p{VT1.2} (red) flies. Data recorded during the last three days of LD for individual flies (for each genotype, n > 50) was combined to obtain the group averages shown. To facilitate comparison, the peak value for evening activity for each genotype was set to 1 and all other values normalized. Grey shading, lights-off.

The relative differences in the daily activity profiles of the p{VT1.1} and p{VT1.2} transgenic flies as a function of temperature were very similar to those observed in the VT1.1 and VT1.2 inbred lines, respectively (compare Figures 2 and 4). Most notably, at 18°C the p{VT1.2} flies exhibit a more robust mid-day siesta compared to p{VT1.1} flies (Figure 4 and Table 1). As expected, at higher temperatures the differences in daily activity profiles between p{VT1.1} and p{VT1.2} flies were less significant due to the direct effects of light on suppressing daytime activity (e.g., [5], [12]). As with the inbred lines, the p{VT1.1} and p{VT1.2} flies have similar free-running periods (Table 2), further supporting the contention that while changes in dmpi8 splicing efficiency modulate daily activity patterns they do not have significant effects on the pace of the clock. One possible difference in the daily activity profiles of the inbred and transgenic lines is that the relative nighttime activity levels are more similar in the transgenics compared to their inbred counterparts (compare figs. 2 and 4), further supporting the notion that dmpi8 splicing efficiency is mainly contributing to daytime activity levels. It is possible that while the efficiency of dmpi8 splicing contributes to differences in mid-day siesta for the inbred VT1.1 and VT1.2 flies, other genetic variations also make contributions to their overall daily activity patterns. In summary, differences in the splicing efficiency of dmpi8 and mid-day siesta observed in the VT1.1 and VT1.2 inbred flies are strongly replicated in transgenic models that solely differ in the type of *dper* 3′ UTR haplotype.

### Splicing Assay in Cultured *Drosophila* Cells Reveals that SNP3 and SNP4 are Important in Regulating dmpi8 Splicing Efficiency

**Figure 5:**
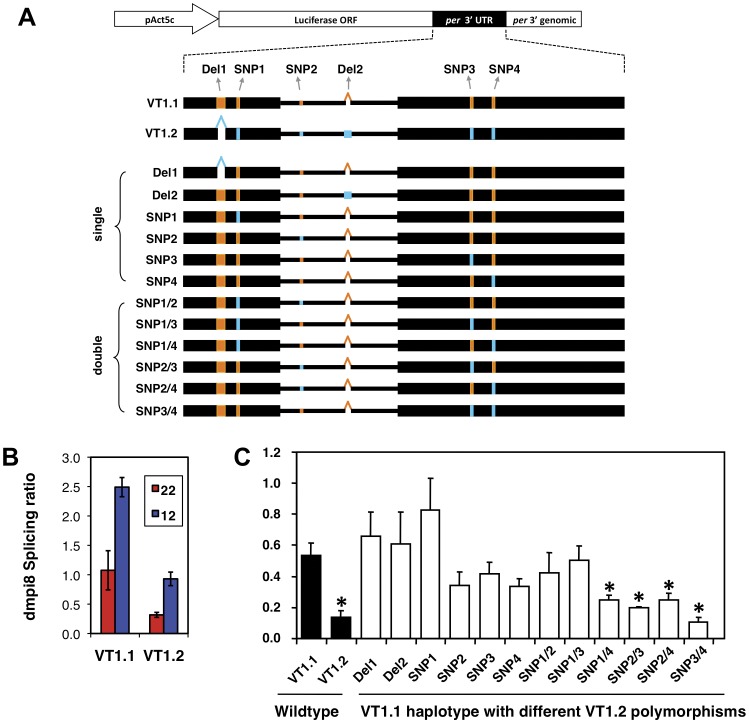
Identification of *dper* 3 ′ **UTR polymorphisms that affect dmpi8 splicing efficiency using** ***Drosophila*** **S2 cells.** **A.** Schematic of plasmids used to evaluate the role of the different *dper* 3′ UTR polymorphisms on dmpi8 splicing efficiency. At the top is shown the outline of the parental *luc*-*dper* 3′ UTR plasmid with key regions depicted, whereas below are shown the different *dper* 3′ UTRs analyzed in this study. The VT1.1 and VT1.2 plasmids contain the wildtype *dper* 3′ UTR sequences found in the VT1.1 and VT1.2 inbred flies, respectively. The VT1.1/VT1.2 hybrids were generated by replacing one (single) or two (double) of the VT1.1 polymorphisms (orange) with the corresponding variant found in VT1.2 (blue). **B, C.** *Drosophila* S2 cells were transfected with the indicated plasmids (bottom of panels), grown at either 12° (B, blue) or 22°C (B, red; panel C), and the ratio of dmpi8 spliced to unspliced mRNA is shown. **C.** *, indicates values that are significantly (p < 0.01, Student *t-test*) different from that of the VT1.1 parental wildtype control. Note; to facilitate comparisons of dmpi8 splicing efficiency, the splicing values are denoted as the ratio between dmpi8 spliced and unspliced transcripts.

To more rapidly evaluate the possible contributions of the different polymorphisms to dmpi8 splicing efficiency, we used a *Drosophila* cell culture system that we previously developed and recapitulates the thermosensitive splicing phenotype of dmpi8 observed in fly heads [3]. In this assay, plasmids based on a *luciferase* (Luc) reporter gene fused to the entire *dper* 3′ UTR including proximal 3′ non-transcribed regions (Fig. 5A) are transfected into *Drosophila* S2 cells that are grown at different temperatures, extracts prepared and dmpi8 splicing efficiency determined using RT-PCR.

In agreement with the *in vivo* results, the splicing efficiency of dmpi8 in the luc-VT1.1 construct was higher compared to the luc-VT1.2 version (Fig. 5B). Furthermore, although the overall splicing efficiency of dmpi8 in VT1.1 is significantly higher compared to VT1.2, it is still enhanced at cold temperatures (Figure 5B), consistent with the thermal sensitivity observed for the splicing of the dmpi8 intron in p{VT1.1} and p{VT1.2} flies (Fig. 3, A and B). This is in sharp contrast to increasing the strengths of the 5′ and 3′ss, which not only increases overall splicing efficiency but also abrogates its thermal sensitivity [3]. Based on the ability to recapitulate the enhanced splicing efficiency of the VT1.1 version compared to the VT1.2 version, we used this cell-based system to systematically evaluate each VT1.1/VT1.2 polymorphism either individually or in combination. To accomplish this we used the VT1.1 *dper* 3′UTR as the parental construct and replaced VT1.1 polymorphisms by the corresponding VT1.2 variants (Fig. 5A). Overall, exchanging a single SNP had small to no effects on the splicing efficiency of dmpi8 in either the VT1.1 or VT1.2 backgrounds (Fig. 5C and data not shown). Among all combinations tested, replacing both SNPs 3 and 4 from the VT1.1 versions to those found in VT1.2 reduced dmpi8 splicing efficiency to a level very similar to that of the VT1.2 parental control (Figure 5C), suggesting SNPs 3/4 play a major role in the differential splicing efficiencies of dmpi8 between VT1.1 and VT1.2 flies.

### SNPs 3 and 4 Regulate dmpi8 Splicing and Daily Activity in Flies

Based on the fact that SNPs 3 and 4 are very close to each other (11 bp apart) and yielded the most striking effects when tested in combination on dmpi8 splicing in the cell-based assay (Fig. 5C), we decided to focus on probing the *in vivo* effects of these two polymorphisms. Thus, we generated transgenic flies bearing the VT1.1 3′ UTR but where SNP3 (termed, p{VT1.1-SNP3}), SNP4 (p{VT1.1-SNP4}) or both (p{VT1.1-SNP3/4}) were replaced with those from VT1.2. At least 3 independent lines of each genotype were obtained and assayed in a *w per* ^01^ genetic background for dmpi8 splicing efficiency and behavioral rhythms.

**Figure 6:**
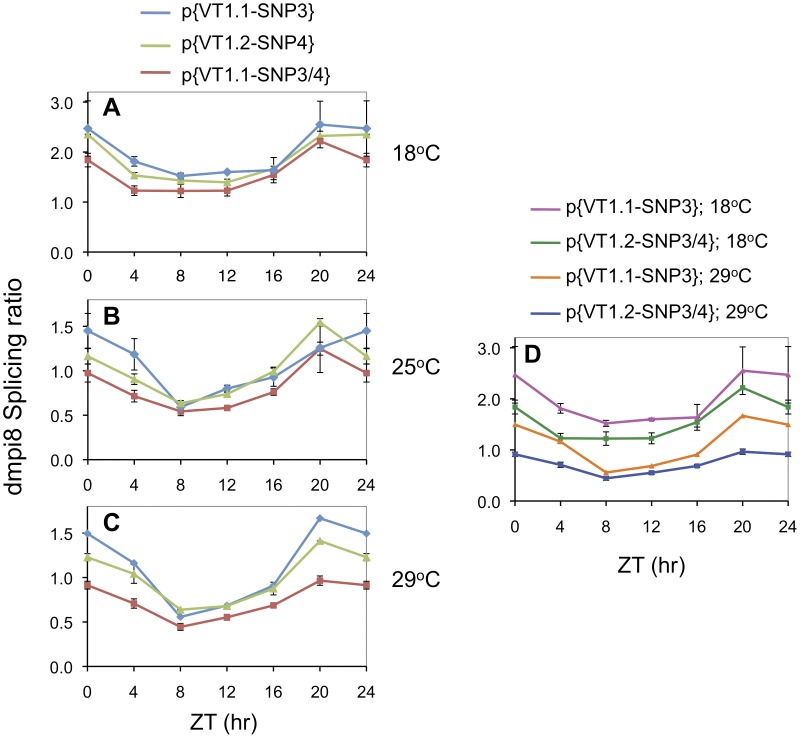
SNPs 3 and 4 from the VT1.2 haplotype reduce the daily splicing efficiency of the dmpi8 intron. A–C. The different transgenic flies, p{VT1.1-SNP3} (blue), p{VT1.1-SNP4} (green) and p{VT1.1-SNP3/4} (red) were exposed to five LD cycles at 18° (A), 25° (B) or 29°C (C). Flies were collected at the indicated times during the last LD, head extracts prepared and shown is the ratio of dmpi8 intron spliced to unspliced *dper* mRNA. **D.** Shown is an overlay of dmpi8 splicing in p{VT1.1-SNP3} and p{VT1.1-SNP3/4} flies at 18° and 29°C (see top of panel) to more easily illustrate that irrespective of the SNP3/4 variant, dmpi8 splicing remains temperature sensitive. For each panel, data from at least three independent experiments were combined to generate the graphs shown. At all temperatures tested, the splicing efficiency for the dmpi8 intron in p{VT1.1-SNP3/4} flies is significantly different from that in either p{VT1.1-SNP3} or p{VT1.1-SNP4} flies (ANOVA, p < 0.01). ANOVA analysis also showed significant temperature effects (p < 0.01) on dmpi8 splicing efficiency for all three genotypes. Note, in this figure the ratio of dmpi8 spliced transcripts to those transcripts where the dmpi8 intron is retained is shown instead of the proportion of *dper* transcripts with dmpi8 spliced, as in figures 2 and 4. Although both methods of plotting yield similar results, using the ratio enhances visualizing the relative differences in the daily dmpi8 splicing curves when comparing these three hybrid genotypes.

At all temperatures tested, the *w per* ^01^;p{VT1.1-SNP3/4} flies (herein, more simply termed p{VT1.1-SNP3/4}) manifested the lowest overall daily splicing efficiency for the dmpi8 intron (Fig. 6A–C), consistent with the results obtained in cultured S2 cells. Based on several experiments it appears that the daily splicing efficiency of dmpi8 is slightly less in p{VT1.1-SNP4} flies compared to p{VT1.1-SNP3} flies, suggesting a more prominent role for SNP4 compared to SNP3. As expected based on results obtained with the VT1.1 and VT1.2 flies, the dmpi8 splicing efficiency in all three VT1.1/1.2 hybrid transgenics (p{VT1.1-SNP3}, p{VT1.1-SNP4}, and p{VT1.1-SNP3/4}) is temperature-dependent (Fig. 6D, and data not shown). Together, these data suggest that SNP3 and SNP4 might function as a unit *in vivo* to regulate the splicing efficiency of dmpi8 with little to no effect on its thermosensitivity.

**Figure 7:**
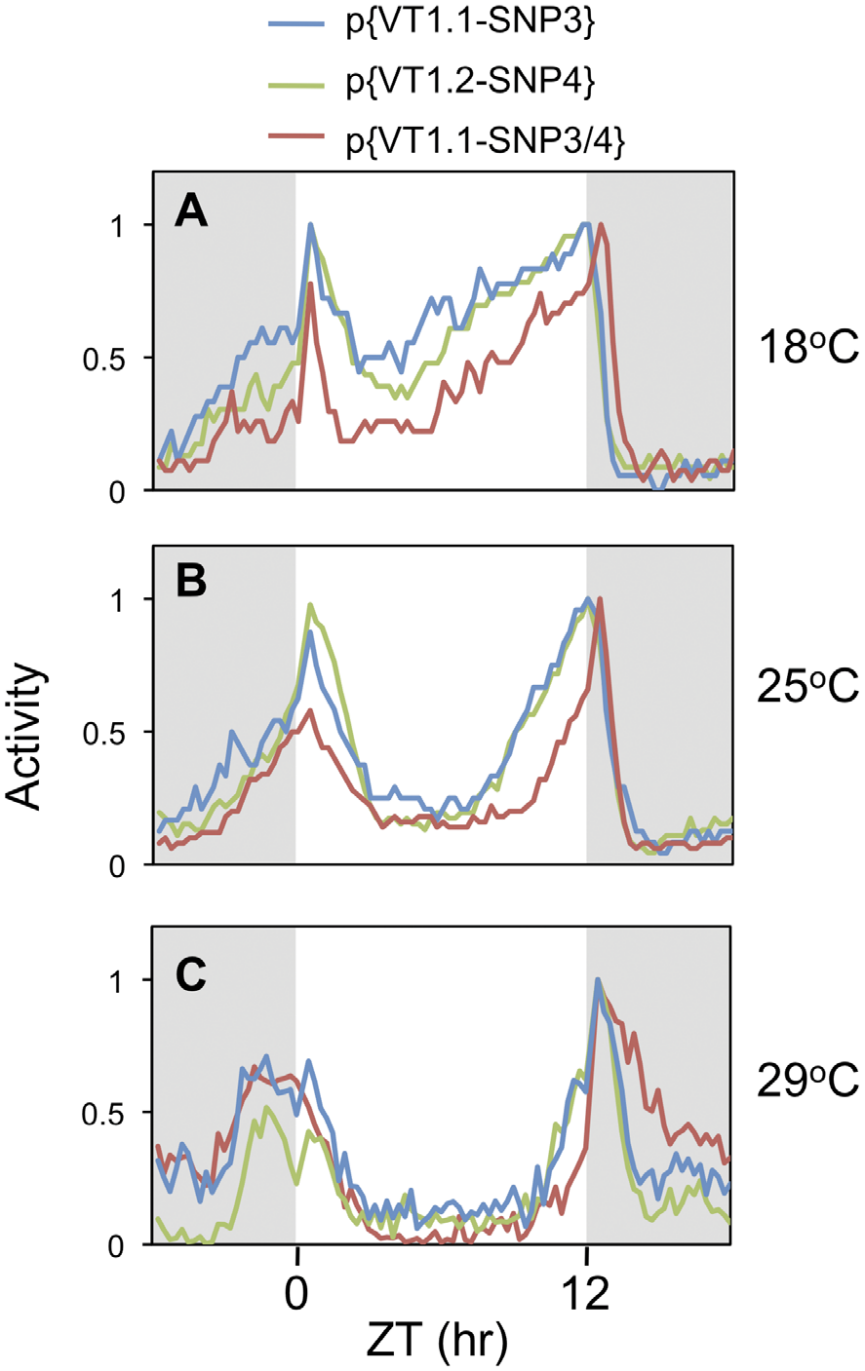
The combination of SNPs 3 and 4 from the VT1.2 haplotype leads to a more robust mid-day siesta and delayed onset of evening activity compared to each SNP alone. Flies were exposed to 12∶12LD cycles for 5 days at 18° (A), 25° (B), or 29°C (C), and then kept in constant darkness (DD) for 7 days. Shown are the locomotor activity profiles for p{VT1.1-SNP3} (blue), p{VT1.2-SNP4} (green), and p{VT1.1-SNP3/4} (red) flies. Data recorded during the last three days of LD for individual flies (for each genotype, n > 50) was combined to obtain the group averages shown. To facilitate comparison, the peak value for evening activity for each genotype was set to 1 and all other values normalized. Grey shading, lights-off.

All three transgenes rescued behavioral rhythmicity in a *w per^0^* genetic background with nearly identical free running periods (Table 2). The mid-day siesta was significantly more robust for p{VT1.1-SNP3/4} flies compared to either p{VT1.1-SNP3} or p{VT1.1-SNP4} (Fig. 7 and Table 1), although as observed in other cases differences were less pronounced at 29°C. As expected, all three transgenic flies showed an increase in mid-day siesta and delayed evening activity with rising temperature. Comparisons between the original parental control lines (i.e., p{VT1.1} and p{VT1.2}) and the more recently generated VT1.1/1.2 hybrids were complicated by the fact that in general the hybrid lines had shorter periods (Table 2), possibly reflecting slight differences in genetic background. Nonetheless, within each of the three groups tested [i.e., 1) inbred, VT1.1 and VT1.2; 2) parental transgenics, p{VT1.1} and p{VT1.2}; and 3) hybrid transgenics, p{VT1.1-SNP3}, p{VT1.1-SNP4} and p{VT1.1-SNP3/4}] the results were highly consistent, wherein the flies with the least efficient daily splicing of dmpi8 exhibited the more pronounced mid-day siestas, especially at the colder temperatures. The only noticeable difference we observed within these three groups is that differences in the mid-day siesta between p{VT1.1-SNP3/4} and the two single hybrids were still readily observed even at higher temperatures such as 25°C (Fig. 7B), although the significance of this, if any, is not presently clear. Thus, we have identified naturally occurring examples of *D. melanogaster* variants that support our original model demonstrating a role for dmpi8 splicing in governing daily activity patterns.

## Discussion

In this study we identified several polymorphisms in the *dper* 3′ UTR from natural populations of *D. melanogaster* that were originally caught along the eastern coast of the United States. Sequencing revealed two major haplotypes for flies from this collection, which are represented by the VT1.1 and VT1.2 variants (Fig. 1A). As an initial approach to investigate the effects of natural variations in the *dper* 3′ UTR on dmpi8 splicing efficiency and/or daily activity patterns we generated VT1.1 and VT1.2 sub-lines from the VT1 isofemale line in an attempt to minimize potential complications from differences in genetic background. Although our studies with these flies were not extensive, the VT.1.1 strain exhibited overall higher daily splicing efficiency for the dmpi8 intron and shorter mid-day siesta compared to the VT1.2 inbred strain (Figs. 1 and 2). As noted before, the relationship between dmpi8 splicing efficiency and mid-day siesta is more readily observed at lower temperatures because as ambient temperature increases, light has more potent effects on directly suppressing activity levels [5], [12]). Importantly, the molecular and behavioural results with the VT1.1 and VT1.2 inbred strains were recapitulated in transgenic models (Figs. 3 and 4), demonstrating that naturally occurring polymorphisms in the 3′ UTR of *dper* can modulate the overall daily splicing efficiency of the dmpi8 intron and the daily distribution of activity. Based on results obtained in a simplified *Drosophila* S2 cell culture system (Fig. 5), we focused on two closely spaced SNPs, termed SNPs 3 and 4 that might function as a unit to regulate dmpi8 splicing. Indeed, introducing the VT1.2 SNPs 3 and 4 into the VT1.1 background (i.e., p{VT1.1-SNP3/4}), resulted in flies with less efficient dmpi8 splicing efficiency and longer mid-day siestas compared to either polymorphism alone (Figs. 6 and 7). Taken together, our findings identify naturally occurring examples of *D. melanogaster* strains that support our earlier model for a role for dmpi8 splicing in regulating daily activity patterns [3], [5].

In earlier work we showed that the splicing efficiency of dmpi8 is temperature dependent, whereby splicing is progressively inhibited as ambient temperature rises [5]. The temperature dependent dmpi8 splicing phenotype is mainly due to suboptimal 5′ and 3′ss [3]. To date we analyzed hundreds of different natural and laboratory strains of *D. melanogaster* from various parts of the world and they all contain the same weak 5′ and 3′ss sequences, consistent with the observation that thermal regulation of dmpi8 splicing and mid-day siesta appear to be a ubiquitous feature of *D. melanogaster* ([3] and data not shown). It was further suggested that the thermal responsiveness of the dmpi8 intron is likely based on reduced binding between the U1 snRNA and the suboptimal 5′ss [3]. By optimizing the dmpi8 5′ and 3′ss we showed that the splicing efficiency of dmpi8 is essentially 100% under a wide range of physiologically relevant temperatures [3]. Moreover, these flies exhibit a much less robust and shorter mid-day siesta compared to their control wildtype counterparts [3].

In contrast, although the average daily splicing efficiency of dmpi8 in p{VT1.1} flies is higher compared to that observed in p{VT1.2} flies, splicing is still highly responsive to temperature (Fig. 3C). This is also the case when we examined the VT1.1/1.2 hybrids (i.e., p{VT1.1-SNP3}, p{VT1.1-SNP4} and p{VT1.1-SNP3/4}) (Fig. 6D). Clearly, the differences in the overall daily splicing efficiency of dmpi8 between the VT1.1 and VT1.2 flies and between the different VT1.1/1.2 hybrids are much less than those previously observed when we optimized the dmpi8 5′ and 3′ss [3]. However, alterations in the splicing efficiency of dmpi8 as a result of variations in *dper* 3′ UTR polymorphisms are still being translated into significant differences in mid-day siesta and the distribution of daily activity (Figs. 2, 4 and 7; Table 1). This suggests that even relatively small changes in overall dmpi8 splicing efficiency are physiologically significant, providing the possibility of an exquisite fine-tuning mechanism for behavioural plasticity in *D. melanogaster*. Thus, the combination of multiple weak splicing signals and variations in 3′ UTR polymorphisms generates a molecular mechanism that is thermally responsive but can be ‘tweaked’ to yield a range of dmpi8 splicing efficiencies, possibly contributing to heritable variations in the daily wake-sleep profiles of *D. melanogaster*.

Based on their positions relative to the dmpi8 intron, it is unlikely that any of the polymorphisms we identified in this study directly affect the intrinsic strengths of the key splicing signals such as the 5′ and 3′ss and the branch point (Fig. 1A). The 5′ and 3′ splice site recognition sequences usually involve some 10–20 nucleotides at the exon/intron junctions, with the majority located within the intron [15]. Of the six polymorphisms we identified in the *dper* 3′ UTR, two are in the dmpi8 intron, SNP2 and DEL1 (Fig. 1A) (DEL1 was originally identified when comparing sequences from the Canton S and Oregon R *D. melanogaster* strains; [16]). Studies in our S2 cell system suggest that DEL1 has little effect on dmpi8 splicing efficiency, whereas SNP2 might make a contribution, especially in combination with other SNPs (Fig. 5C). Further studies using transgenic models will be required to more extensively probe the physiological significance of the polymorphisms we identified. With regards to SNPs 3 and 4, they are located more than 70 bp downstream of the 3′ss (Fig. 1A). While speculative, it is possible that natural polymorphisms in the *dper* 3′ UTR, such as SNPs 3 and 4, affect dmpi8 splicing by functioning as binding sites for *trans-*acting factors such as SR-proteins involved in the regulation of alternative splicing [17]. The fact that replacing a single SNP at a time seems to have little effect on dmpi8 splicing (Figs. 5), suggests interaction between the different SNPs.

Intriguingly, the *dper* 3-terminal intron from several *Drosophila* species that are indigenous to Afro-equatorial regions have strong 5′ and 3′ss [3]. In these species, the 3′-terminal intron splicing efficiency and daily activity patterns show little to no temperature regulation, suggesting the suboptimal splices sites on the dmpi8 intron contributed to the colonization of temperate regions by *D. melanogaster* [3]. Indeed, we and others proposed that the dmpi8 thermosensitivity is part of a seasonal, or more precisely thermal, adaptation program in *D. melanogaster* [2], [3], [4], [5]. For example, on warm days the inefficient splicing of dmpi8 leads to a more robust mid-day siesta, presumably minimizing the deleterious effects associated with being active during the hot mid-day sun, such as desiccation. Conversely, on cold days the enhanced splicing of dmpi8 leads to preferential daytime activity, presumably as a means to maximize activity during the warmer daytime hours. Clearly, under standard laboratory conditions of daily light/dark cycles at constant temperatures, the relationship between dmpi8 splicing efficiency and mid-day siesta is not as strong at higher temperatures because light can directly suppress activity whereas dmpi8 splicing efficiency retains its thermal sensitivity. It is possible that in the wild, differences in dmpi8 splicing efficiency (such as those found in the VT1.1 and VT1.2 strains) are mainly rate-limiting for shaping the distribution of daily activity during cooler days where the effects of light intensity on activity levels are not as dominant. In this regard, future studies will be required to determine if the polymorphisms we identified in the 3′ UTR of *dper* provide any fitness advantage to *D. melanogaster* in the wild. To date we did not find evidence of natural selection or geographical variation for any of the *dper* 3′ UTR polymorphisms that we analyzed (data not shown). However, it is possible that this is an idiosyncrasy of the flies we used, which were limited to the Atlantic coast of the United States.

A recent study has questioned the physiological significance of the temperature modulated mid-day siesta in *D. melanogaster* [18]. Rather, they found that in the presence of daily fluctuations in temperature and light intensity, *D. melanogaster* exhibit a mid-afternoon peak in activity. This ‘afternoon’ bout of activity appears unrelated to dmpi8 splicing and is modulated by but not dependent on a functional clock. Further work will be required to elucidate the possible physiological contributions of a mid-day siesta or afternoon activity on the wake-sleep cycles of *D. melanogaster* in nature where they can roam freely. Nonetheless, our results clearly demonstrate that, at least under laboratory conditions, naturally occurring polymorphisms in the 3′ UTR of the central clock gene *dper* can modulate daily activity patterns in a manner consistent with our earlier model showing that dmpi8 splicing functions as a temperature regulated molecular sensor in the thermal adaptation of behaviour in *D. melanogaster*.

## Materials and Methods

### Fly Strains and General Handling

All flies were routinely reared at room temperature (22–25°C) and maintained in vials or bottles containing standard agar-cornmeal-sugar-yeast-Tegosept-media. The generation of transgenic flies is described below. The natural populations of *D. melanogaster* used in this study were obtained from Dr. Eanes (State University of New York, Stony Brook, New York) and are described in Verrelli and Eanes (2001). Briefly, independent isofemale lines were established from flies captured from each of 10 populations along the Atlantic coast of the United States in 1997. The inbred lines termed VT1.1 and VT1.2 were generated from the *D. melanogaster* VT97.1 isofemale line, originally captured in Whiting, Vermont as previously described [9]. DNA sequencing indicated the presence of two *dper* 3′ UTR haplotypes present in this strain. Single-pair crosses were performed to generate inbred strains homozygous for either of the two haplotypes. Four sub-lines were recovered from each of the two kinds of *dper* 3′ UTRs, as confirmed by DNA sequencing. Inbred strains of the same *dper* 3′ UTR haplotype were then pooled to form two inbred lines, termed VT1.1 and VT1.2 respectively, in order to minimize health issues that could arise from excessive inbreeding. In addition, the VT1.1 and VT1.2 lines were maintained in large bottles.

### Transformation Constructs and Transgenic Flies

To generate transgenic flies that produce *dper* transcripts with either the VT1.1 or VT1.2 3′ UTR we used a CaSpeR-4 based transformation vector containing a 13.2 kb genomic *dper* insert [13], [16] that was modified by inserting sequences for KpnI and ApaI sites just upstream of the *dper* translation stop signal, herein termed CaSpeR13.2-KA. The KpnI and ApaI sites are unique in the plasmid, facilitating the subcloning of different 3′ UTRs. Genomic DNAs from VT1.1 or VT1.2 inbred flies were used as templates to amplify the *dper* 3′ UTR sequences with the primers KpnI_P6869 (5′-TAA***GGTACC*** TAGTAGCCACACCCGCAGT-3′) and P7373 (5′- GTGGGCGTTGGCTTTTCG-3′) (the KpnI site is in bold italic, immediately upstream of the two TAG translation stop codons found on wildtype *dper*; genomic DNA sequences are underlined). This yields a DNA fragment from the *dper* stop codon at position 6868 to nucleotide 7373 in the *dper* 3′ UTR (numbering according to Citri et al., 1987). The PCR products were then ligated to the pGEM-T easy vector (Promega) and confirmed by sequencing. Subsequently, a DNA fragment containing sequences from either the VT1.1 or VT1.2 3′ UTR was inserted into the CaSpeR13.2-KA backbone by swapping the DNA fragment from the Kpn1 site to a unique Bsu36I site in the *dper* 3′UTR to yield the final transgenic constructs, CaSpeR13.2-KA/VT1.1 and CaSpeR13.2-KA/VT1.2. The entire *dper* 3′ UTR is ∼500 nt long (depending on the haplotype) and the Bsu36I site is ∼70 bp upstream of the site for 3′ cleavage/polyadenylation; note, the VT1.1 and VT1.2 *dper* 3′ UTR sequences are identical downstream of the Bsu36I site (data not shown).

To examine the physiological significance of SNP3 and SNP4 we utilized the pGEM/VT1.1 construct to substitute SNP3 and/or SNP4 with those found in VT1.2 by using the Quick Change site-directed mutagenesis kit (Stratagene, CA, USA). Subsequently, the different *dper* 3′ UTRs were reconstructed into the CaSpeR13.2-KA/VT1.1 transformation vector (see above), to yield the final constructs (CaSpeR13.2-KA/VT1.1-SNP3, CaSpeR13.2-KA/VT1.1-SNP4, CaSpeR13.2-KA/VT1.1-SNP3/4).

Relevant regions of the transformation vectors were confirmed by sequencing before delivery to Genetic Services Inc (Sudbury, MA, USA) for injection into a *w* ^1118^ background. Recovered germ-line transformants were subsequently crossed into a *w per* ^0^ background with a double balancer line (*w per* ^0^;*Sco*/*Cyo*;*MKRS*/*TM6B*), resulting in the transgenic lines termed; *w per* ^0^;p{VT1.1}, *w per* ^0^;p{VT1.2}, *w per* ^0^;p{VT1.1-SNP3}, *w per* ^0^;p{VT1.1-SNP4} and *w per* ^0^;p{VT1.1-SNP3/4}. Thus, the only functional version of *dper* in these transgenic flies is the transgene. At least three independent lines for each construct were obtained. The results shown in this manuscript were largely derived by pooling data from the following lines: p{VT1.1}, f11f, m36mo, m45m; p{VT1.2}, f43, m1m7, m3m1; p{VT1.1-SNP3}, f36m1b, f5f1, f28m3b; p{VT1.1-SNP4}, m23m1b, f7m1b, m43m3b, m44m1b; p{VT1.1-SNP3/4}, m10m4, m17f1, m21f1.

### Locomotor Activity

Locomotor activity was continuously monitored and recorded in 15-min or 30-min bins by placing individual adult male flies (three to seven day-old males) in glass tubes and using a Trikinetics (Waltham, MA, USA) system, as previously described [1], [19]. Briefly, throughout the testing period flies were maintained at the indicated temperature (18°, 25° or 29°C) and subjected to at least 5 days 12 hr light: 12 hr dark cycles [LD; where zeitgeber time 0 (ZT0) is defined as lights-on], followed by 7 days of constant dark conditions (DD). Cool white fluorescent light (∼2000 lux) was used during LD and the temperature did not vary by more than 0.5°C between the light and dark periods. For measuring behavioural values during LD cycles, at least two days worth of data were averaged for each fly, and data from different lines pooled to generate the group average for each genotype. Data analysis was done on a Macintosh computer with the FaasX software (kindly provided by M. Boudinot and F. Rouyer, CNRS, France), which is based on the Brandeis Rhythm Package (originally developed in the laboratories of J. Hall and M. Rosbash, Brandies University, MA, USA). Free-running periods and power (amplitude or strength of the rhythm) were obtained using the *Chi*-square periodogram module available within the FaasX program using activity data collected in 30 min bins during at least 5 consecutive days in DD. Flies with power ≥10, width ≥2, and periods between 20–30 hr were designated rhythmic. Values for individual flies were pooled to obtain an average value for each genotype.

The timing of morning and evening peaks, 50% morning offset and 50% evening onset were determined on a Unix command line version of the Brandeis Rhythm Package (BRP) Phase module, as previously described [3]. Similar results were obtained when we varied the onset and offset phase reference points from 25 to 75% of peak values (data not shown), and results with 50% are shown as they were the most reproducible. The values for morning/evening onset/offset were based on pooling data from multiple individual flies over the last three days of LD using data collected in 30 min bins. The mid-day siesta is defined as the time between 50% morning offset and 50% evening onset, as previously described [3]. ANOVA and appropriate post-hoc analysis were performed using SPSS 16.0 (SPSS Inc., Chicago, USA). Daily locomotor activity profiles were normalized such that the peak of evening activity was set to 1, facilitating visual comparison of the different transgenic genotypes, as previously described [3]. A correction was applied to neutralize “startle responses” (i.e., transient increased bout of fly activity immediately following the light-to-dark and/or dark-to-light environmental transitions) [11]; essentially the activity counts in the bin right after the environmental transition is replaced by an average of the activity counts in the bins just before and after.

### Tissue Culture Constructs and dmpi8 Splicing Assay

A previously described construct [3], which contains the pAct5c promoter fused to the *luciferase* (*luc*) open reading frame followed by the entire *dper* 3′ UTR and approximately 90 bp of 3′ *dper* non-transcribed region (termed pAct-Luc-*dper*3′UTR, or more simply termed 8∶8) was used as the backbone vector to swap the VT1.1 and VT1.2 *dper* 3′ UTRs. To generate pAct-Luc-VT1.1 and pAct-Luc-VT1.2 constructs, genomic DNA from either VT1.1 or VT1.2 inbred flies was used as a template to amplify the *dper* 3′ UTR with the primers StuI_P6869 (5′-TA***AGGCC*** TAGTAGCCACACCCGCAGT-3′) and P7373 (5′- GTGGGCGTTGGCTTTTCG-3′) (The StuI site is in bold italic, immediately upstream of the *dper* translation stop codon; genomic DNA sequences are underlined.). The VT1.1 and VT1.2 3′ UTRs were reconstructed into the pAct-Luc-*dper*3′UTR vector by swapping the DNA fragment from the StuI site to the Bsu36I site in the *dper* 3′ UTR (see above, for transformation constructs) to yield pAct-Luc-VT1.1 and pAct-Luc-VT1.2. To generate plasmids containing the VT1.1 3′ UTR but wherein one or more VT1.1 polymorphisms were replaced by the cognate VT1.2 version, we first subcloned the VT1.1 3′ UTR into the pGEM-T Easy vector (Promega, USA), which was used as a basis for mutagenesis using the Quick Change site-directed mutagenesis kit (Stratagene, CA, USA). Subsequently, the different VT1.1/VT1.2 hybrid versions were reconstructed into the pAct-Luc-VT1.1 vector by swapping the DNA fragment from StuI site to the Bsu36I site in the *dper* 3′ UTR. All final constructs used in this study were validated by DNA sequencing prior to their further use.

Measurement of the dmpi8 splicing efficiency in *Drosophila* S2 cells was performed essentially as previously described [3]. Briefly, the S2 cells and DES expression medium were purchased from Invitrogen. S2 cells were transiently transfected using Effectene reagent (Qiagen), according to manufacturer’s instructions. Approximately 1.5×10^6^ S2 cells were placed in 6-well plates and transfected with 125 ng of the different pAct-Luc-UTR vectors. After transfection, cells were allowed to recover for 2 days. Subsequently, cells were transferred to the indicated temperature for overnight incubation before harvesting. Cells were collected and washed twice with PBS on ice. Total RNA was extracted using the TRI Reagent LS (Sigma) according to manufacturer’s instructions. For each sample, about 1 µg of RNA was subjected to reverse transcription-PCR (RT-PCR) and dmpi8 splicing efficiency measured in the presence of the forward primer P6869 and the reverse primer P7197, as previously described [3], [4].

### Splicing Assay in Flies

The splicing efficiency of dmpi8 in flies was measured as previously described [3], [4], with some minor modifications. Briefly, vials containing ∼100 young (2- to 6-day-old) adult flies were placed in environmental chambers (Percival, USA) at the indicated temperature and exposed to at least five 12 hr light: 12 hr dark cycles. At selected times during LD, flies were collected by freezing and heads isolated. Total RNA was extracted and the relative levels of *dper* mRNA either containing the dmpi8 intron or in which it was spliced measured using a semi-quantitative RT-PCR assay as previously described [3]. In order to differentiate between the transgenic derived *dper* mRNA transcripts from the endogenously derived *per* ^01^ transcripts we used the forward primers P6851m2F (5′-ACAGCACGGGGATGGG***GGTACC***-3′; KpnI site is in bold and italicized) and P6851 (5′ ACACAGCACGGGGATGGGTAGT 3′), respectively. The P6851 primer will only amplify endogenously derived *dper* mRNA (e.g., *per* ^01^ mRNA), whereas the P6851m2F primer will only amplify transgenic derived *dper* transcripts that contain the engineered Kpn1 site upstream of the *dper* translation stop codon. All RT-PCR reactions included the *dper* P7197 reverse primer; and the forward primer CBP540F (5′ GTCTGATTCGTGTGGACTGG 3′) and reverse primer CBP673R (5′ CAACAGTTTGCCATAACCCC 3′) to target the non-cycling Cap Binding Protein 20 (CBP20) gene as an internal control, as previously described [3], [4]. PCR products were separated and visualized by electrophoresis on 2% agarose gels containing Gelstar (Cambrex Co., USA), and the bands quantified using a Typhoon 9400 Imager. The values of *dper*-containing amplified products were normalized relative to CBP20 and are routinely expressed as either the proportion of the total *dper* mRNA with the 3′-terminal intron removed, or the relative ratio of dmpi8 spliced transcripts to those that are unspliced. The relative abundance of total *dper* mRNA was calculated by adding the values for the two RT-PCR products; i.e., with and without the dmpi8 intron. We routinely collected samples after different PCR cycle lengths to ensure that the amplified products were in the linear range for quantification.
